# Supplementary material for: Do skin bacteriostatic agents reduce acute radiodermatitis in breast cancer patients? A prospective interventional study
Source: PLoS One. 2025 Aug 6;20(8):e0328536. doi: 10.1371/journal.pone.0328536 (PMC12327638; doi:10.1371/journal.pone.0328536)
Supplement: S1 File — (PDF) [file pone.0328536.s001.pdf]

No.

Name：		Type of surgery：	ALND：Y/N
Radiotherapy dose：	Number of radiotherapy：	Radiation: Hypofractionation/Regular fractionation	Site and dose of radiotherapy：
Age：	Smoking： Y/N	Height：	Weight：
Diabetes：Y/N	Ultraviolet allergy：Y/N	Skin type：	

Radiodermatitis Record Card

Number of radiotherapy	5	10	15	20	25	One week after radiotherapy	Two week after radiotherapy
Skin protectant							
Dermatitis grade (RTOG)							
Self-reported symptoms							
